# Supplementary figures and images for: Translation of unspliced retroviral genomic RNA in the host cell is regulated in both space and time
Source: J Cell Biol. 2025 Jan 27;224(4):e202405075. doi: 10.1083/jcb.202405075 (PMC11775842; doi:10.1083/jcb.202405075)

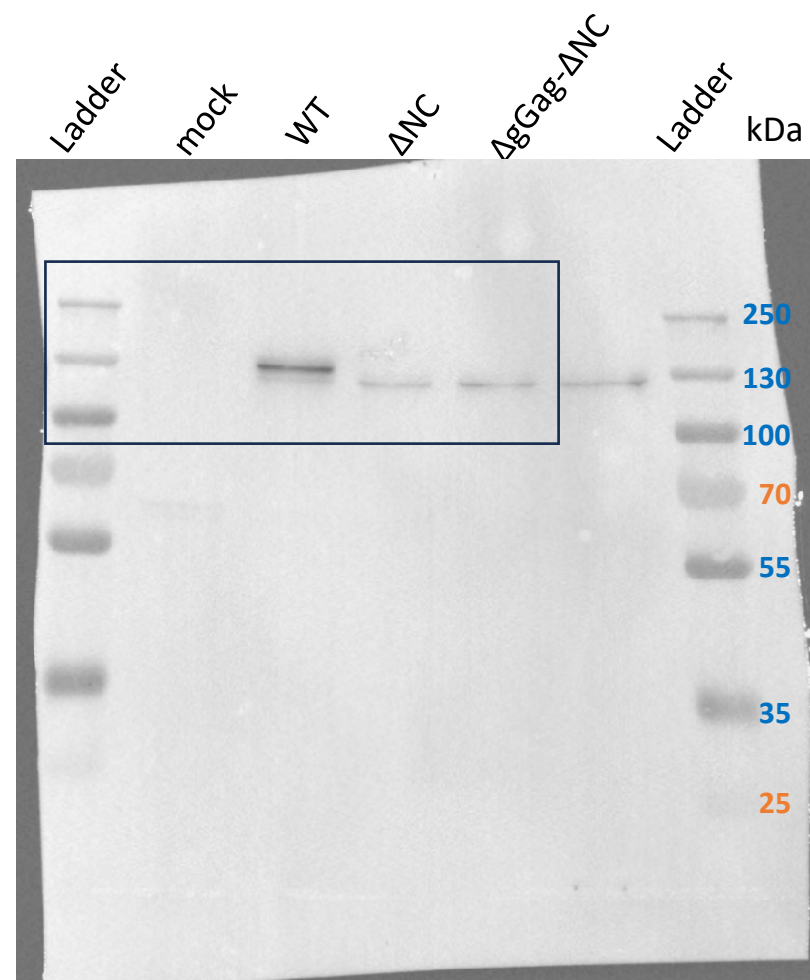

Supplement: SourceData FS1 — is the source file for Fig. S1. [file jcb_202405075_sourcedatafs1.pdf]
